# Machine-learning-assisted integration of single-cell and bulk transcriptomics characterizes glioblastoma ecosystem states and immune-associated programs

**DOI:** 10.3389/fcell.2026.1967483

**Published:** 2026-10-14

**Authors:** Jun Cao, Hao Cheng, Yu Wang, Bing Zhao, Yang Wang, Xiaochun Jiang

**Affiliations:** 1 Department of Neurosurgery, The Second Affiliated Hospital of Anhui Medical University, Anhui Medical University, Hefei, China; 2 Department of Neurosurgery, The First Affiliated Hospital of Wannan Medical University (Yijishan Hospital of Wannan Medical University), Wuhu, China; 3 Department of Neurosurgery, Shanghai Ninth People’s Hospital, Shanghai Jiao Tong University School of Medicine, Shanghai, China; 4 Department of Neurosurgery, The First Affiliated Hospital of Wannan Medical University (Yijishan Hospita), Wuhu, China

**Keywords:** glioblastoma, machine learning, malignant cell states, RT-qPCR, single-cell RNA sequencing, tumor ecosystem, tumor microenvironment

## Abstract

**Background:**

Glioblastoma (GBM) is characterized by marked malignant-cell plasticity and a complex tumor ecosystem composed of heterogeneous malignant and microenvironmental cell populations. We integrated single-cell and bulk transcriptomic data to characterize reproducible GBM cell and ecosystem-associated states, evaluate cross-platform concordance, and examine immune-associated transcriptional programs while distinguishing descriptive associations from mechanistic inference.

**Methods:**

The deposited Smart-seq2 (7,930 cells) and 10x (16,201 cells) expression matrices from GSE131928/SCP393 were analyzed on their verified expression scales. Official annotations and malignant-state modules were audited, four broad malignant states were recomputed, and a tumor-held-out machine-learning label-transfer framework was applied to the 10x dataset. Public TCGA-GBM RNA-seq data were used to evaluate state- and microenvironment-associated transcriptional programs and to perform nested prediction benchmarking. Selected ecosystem-associated genes were additionally examined by RT-qPCR in glioma cell lines and normal human astrocytes.

**Results:**

Among Smart-seq2 cells, 6,576 malignant cells had valid state scores, whereas 287 alias-affected records were excluded from state-score denominators. The machine-learning label-transfer model achieved an out-of-fold accuracy of 0.998, and malignant-state fractions showed moderate-to-strong concordance across six shared tumor labels. At the tumor-label level, MES-like fraction was not significantly associated with macrophage fraction (Spearman rho = 0.171, P = 0.395), indicating that ecosystem-level transcriptional association should not be interpreted as a simple directional immune-to-state mechanism. In TCGA-GBM, MES and myeloid-associated programs correlated with immune and stromal expression signatures, whereas survival and 12-month prediction performance remained modest and specification-sensitive. RT-qPCR further demonstrated increased STAT3, CHI3L1, SPP1, and VEGFA expression in glioma cell lines relative to normal human astrocytes.

**Conclusion:**

Integrated single-cell and bulk transcriptomic analysis supports reproducible characterization of malignant-cell heterogeneity and immune-associated ecosystem features in IDH-wildtype GBM. Machine-learning-assisted cross-platform analysis provides a reproducible framework for evaluating tumor ecosystem-associated states, while the observed immune-mesenchymal associations remain descriptive rather than causal. Experimental RT-qPCR provides expression-level support for selected ecosystem-associated genes and highlights testable hypotheses for future functional and spatial studies.

## 1 Introduction

Glioblastoma (GBM), defined in the 2021 World Health Organization classification as an IDH-wildtype diffuse astrocytic glioma with characteristic histologic or molecular features, is the most aggressive common primary malignant brain tumor in adults (Louis et al., 2021; Ostrom et al., 2022). Despite maximal safe resection followed by radiotherapy and temozolomide-based chemotherapy, outcomes remain poor (Stupp et al., 2005). Contemporary molecular classification has improved diagnostic precision, but it does not eliminate the substantial intratumoral transcriptional heterogeneity that complicates biological interpretation and therapeutic development (Cancer Genome Atlas Research Network, 2008; Brennan et al., 2013; Verhaak et al., 2010; Brat et al., 2015).

Single-cell RNA sequencing has shown that malignant GBM cells occupy multiple recurrent transcriptional states rather than a single fixed subtype. In the Neftel et al. framework, malignant cells can be organized around astrocyte-like (AC-like), mesenchymal-like (MES-like), neural-progenitor-like (NPC-like), and oligodendrocyte-progenitor-like (OPC-like) programs, with considerable within-tumor coexistence and plasticity (Neftel et al., 2019). Other single-cell studies similarly demonstrate extensive heterogeneity across malignant and non-malignant compartments (Darmanis et al., 2017; Patel et al., 2014; Wang et al., 2019; Richards et al., 2021). These observations support a view of GBM as a dynamic cellular system in which tumor-intrinsic programs and the surrounding microenvironment jointly shape the observed transcriptome (Hanahan, 2022).

The GBM microenvironment contains abundant myeloid cells, including resident microglia and recruited macrophage populations, together with lymphoid and vascular compartments (Klemm et al., 2020; Pombo Antunes et al., 2021). T-cell dysfunction is also prominent in GBM (Woroniecka et al., 2018). However, correlations between immune abundance and malignant-cell state do not by themselves establish directional signaling or causal state transitions. This distinction is especially important when analyses aggregate cells at the tumor-label or bulk-RNA level, where cellular composition, tumor purity, and shared transcriptional programs can generate strong expression-level associations.

Here, we used an ecosystem-oriented integrative framework to reanalyze the public GSE131928/SCP393 dataset generated by Neftel et al., comprising 24,131 single cells from IDH-wildtype GBM profiled by Smart-seq2 and 10x platforms (Neftel et al., 2019). We combined malignant-cell state reconstruction, tumor microenvironment characterization, machine-learning-assisted cross-platform label transfer, and bulk TCGA-GBM transcriptomic analysis to evaluate the reproducibility and clinical relevance of GBM ecosystem-associated transcriptional states. Selected genes representing malignant, mesenchymal/myeloid, and angiogenic programs were further examined by RT-qPCR in glioma cell lines and normal human astrocytes. This design was intended to connect computational ecosystem modeling with experimentally derived biological data while maintaining a clear distinction between transcriptional association and functional or causal validation.

## 2 Methods

### 2.1 Data provenance and disease matching

The single-cell input was GSE131928/SCP393, an IDH-wildtype GBM study linked to BioProject PRJNA545332. The bulk input was TCGA-GBM. Official GEO, BioProject, Single Cell Portal, PubMed, and GDC records were checked against local file contents. The local single-cell metadata contains 28 Smart-seq2 tumour labels and 9 10x labels, with six exact labels shared across platforms; the official study summary of 28 tumours was not reinterpreted as 31 independent patients.

### 2.2 Single-cell scale and annotation audit

The Smart-seq2 matrix was used as deposited on the log_2_ (TPM/10 + 1) scale. The 10x matrix was verified as TPM-like linear data and transformed once to log_2_ (TPM/10 + 1). Official SCP393 cell assignments, tSNE coordinates, state-hierarchy coordinates, gene-detection values, and six state modules were joined by exact cell identifiers. The 287 malignant records with alias-corrupted all-zero state modules were retained for audit plots but excluded from valid state-score denominators and dominant-state summaries after deterministic identifier correction. No raw FASTQ data were supplied, so alignment and Cell Ranger reprocessing were outside scope.

### 2.3 Label transfer, states, and concordance

Canonical marker families were summarized from the Smart-seq2 matrix. A grouped five-fold logistic model transferred four broad cell labels to 10x cells, with tumour labels held out as groups; performance was reported from out-of-fold predictions. Four collapsed malignant states were recomputed from the official module definitions. Tumour-label fractions, entropy, six-label state correlations, and malignant pseudobulk correlations were calculated only at the stated statistical unit. The 10x embedding used real transformed expression, deterministic PCA, and a fixed-seed UMAP representation.

### 2.4 TCGA cohort and fixed scores

TCGA raw integer counts were normalized to CPM using all genes in each selected library and transformed to log_2_ (CPM + 1). The cohort contract required primary tumour aliquots, paper IDH status WT, one selected aliquot per patient, and complete paper OS fields where survival was analyzed. Fixed state scores were computed from the supplied IDHwt.GBM.MetaModules.tsv gene lists; immune-associated programs were predefined in the analysis code before outcome analysis. Each score records its gene coverage in the output tables.

### 2.5 Differential expression and pathway description

A median split of the fixed MES/immune score was used for a Welch contrast. Benjamini–Hochberg false-discovery control was applied to the gene-level table. Hallmark preranked GSEA used the cached MSigDB Hallmark 2020 library and 1,000 gene-set permutations. Both analyses are explicitly labeled downstream descriptive analyses because the grouping variable is derived from the expression programs under discussion.

### 2.6 Survival and prediction

Overall survival used the paired paper survival-months and paper vital-status fields. Kaplan-Meier curves used the fixed median split, and Cox models used standardized predictors with proportional-hazards diagnostics. The prediction endpoint was 12-month mortality; patients censored at or before 12 months without an event were excluded. Candidate clinical, signature, and combined models used five repeats of five outer patient-level stratified folds and four-fold inner tuning, with fold-local imputation and scaling. Performance was summarized from held-out predictions with 2,000 bootstrap replicates, calibration, decision-curve, and held-out permutation-importance diagnostics, reported with conservative prediction-model conventions.

### 2.7 Reproducibility and statistical conventions

All biological values are read from the supplied or generated result tables; plotting scripts do not recompute hypothesis tests or create biological observations. P values are two-sided unless a table states otherwise, and correlation estimates are interpreted with their sample size and confidence interval. The complete output includes input hashes, scale registries, contracts, panel tables, figure hashes, software versions, and quality-assurance JSON reports.

### 2.8 *In vitro* RT-qPCR validation of selected genes

U87MG, LN229, U251, and T98G glioma cells and normal human astrocytes (NHA) were used for *in vitro* RT-qPCR validation. Total RNA was isolated from three independent biological cultures per cell type using a phenol/guanidinium- or silica-column-based method and was accepted for analysis when A260/280 was 1.8–2.1 and RNA integrity was visually adequate. One microgram of total RNA was reverse-transcribed in a 20-µL reaction using random hexamer/oligo(dT) priming according to the reverse-transcription reagent instructions. Quantitative PCR was performed with SYBR Green chemistry in 10–20-µL reactions containing cDNA, gene-specific primers, and 2x master mix. The cycling program consisted of an initial denaturation step followed by 40 amplification cycles and a melt-curve analysis to confirm a single predominant amplicon. Each biological replicate was analyzed in three technical wells. STAT3, CHI3L1, SPP1, and VEGFA were quantified relative to GAPDH and ACTB; the geometric mean of the two reference-gene Ct-derived quantities was used for normalization. Relative expression *versus* NHA was calculated using the 2^-DeltaDeltaCt method (Livak and Schmittgen, 2001), and reporting principles followed the MIQE recommendations (Bustin et al., 2009). Data are presented as mean ± SEM of three biological replicates. Two-sided unpaired t-tests were used for each glioma cell line *versus* NHA, with P < 0.05 considered statistically significant.

## 3 Results

### 3.1 Verified same-disease input and platform boundaries

Figure 1 fixes the verified input boundary. Panel A groups the included GSE131928/SCP393 files into four verified roles, with the two expression matrices contributing 669 of the 674 MB measured footprint. Panel B contrasts the platform-specific composition: 7,930 Smart-seq2 cells carrying 28 tumour labels on the deposited log_2_ (TPM/10 + 1) scale, and 16,201 10x cells carrying 9 tumour labels transformed once from TPM-like values. Panel C resolves exact string-matched membership into 22 Smart-seq2-only, 6 shared, and 3 10x-only tumour labels, so all cross-platform work was restricted to the shared labels. Panel D shows the resulting sampling depth, with a median of 266.5 Smart-seq2 *versus* 1,613 10x cells per tumour label. Panel E confirms that official GenesExpressed detection varies across cell assignments in the deposited annotation. Panel F separates 6,576 valid malignant state-scored cells from 287 alias-affected all-zero records and 1,067 non-malignant cells, fixing the valid state-score denominator used throughout.

**FIGURE 1:**
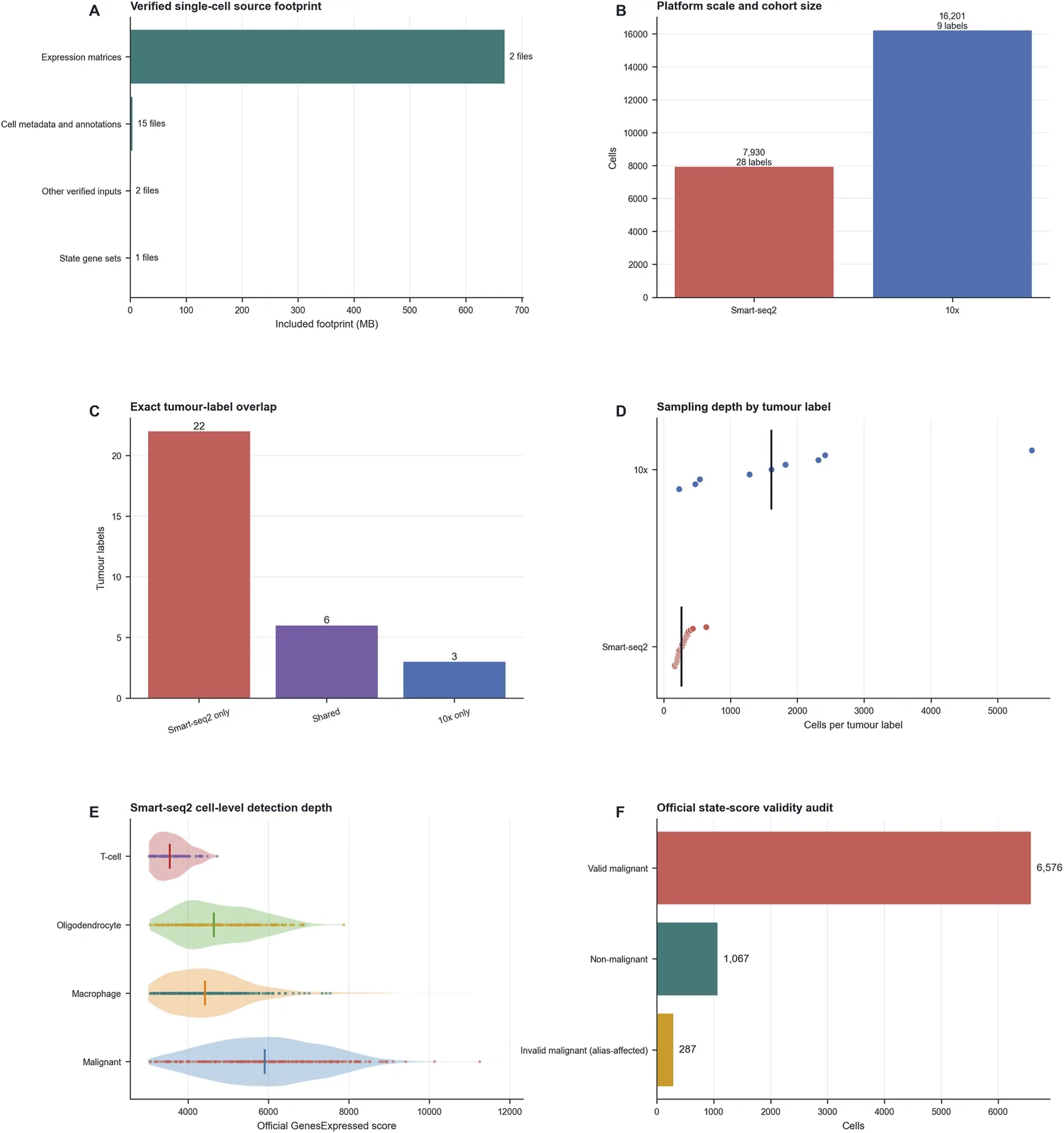
Verified data footprint and single-cell quality boundaries. **(A)** included GSE131928/SCP393 files grouped by verified role and measured file footprint. **(B)** platform-specific cell counts, tumour-label counts, and expression-scale handling; Smart-seq2 was already log_2_ (TPM/10 + 1), whereas 10x was transformed once from TPM-like values. **(C)** exact tumour-label membership across platforms; labels are not relabeled as patients. **(D)** observed sampling depth by tumour label. **(E)** official GenesExpressed values by supplied cell assignment. **(F)** valid *versus* alias-affected malignant state-score records and non-malignant records. All values are copied from the panel tables; the unit is stated in each panel contract.

### 3.2 Single-cell landscape of malignant and ecosystem-associated cell states

Figure 2 layers the deposited annotations on the atlas. Panel A shows the official cell assignments in the published tSNE, Panel B places the supplied adult and pediatric labels on the same coordinates, and Panel C shows that GenesExpressed detection varies across the map. Panel D arranges valid malignant cells in the published state-hierarchy coordinates, colored by the collapsed AC-like, MES-like, NPC-like, and OPC-like states. Panel E summarizes 38 canonical marker genes as mean expression and detection fractions and remains consistent with the supplied broad assignments. Panel F reports the observed quantiles of the six official state-module scores among valid malignant cells. These panels are observed annotation layers rather than evidence for a new unsupervised taxonomy.

**FIGURE 2:**
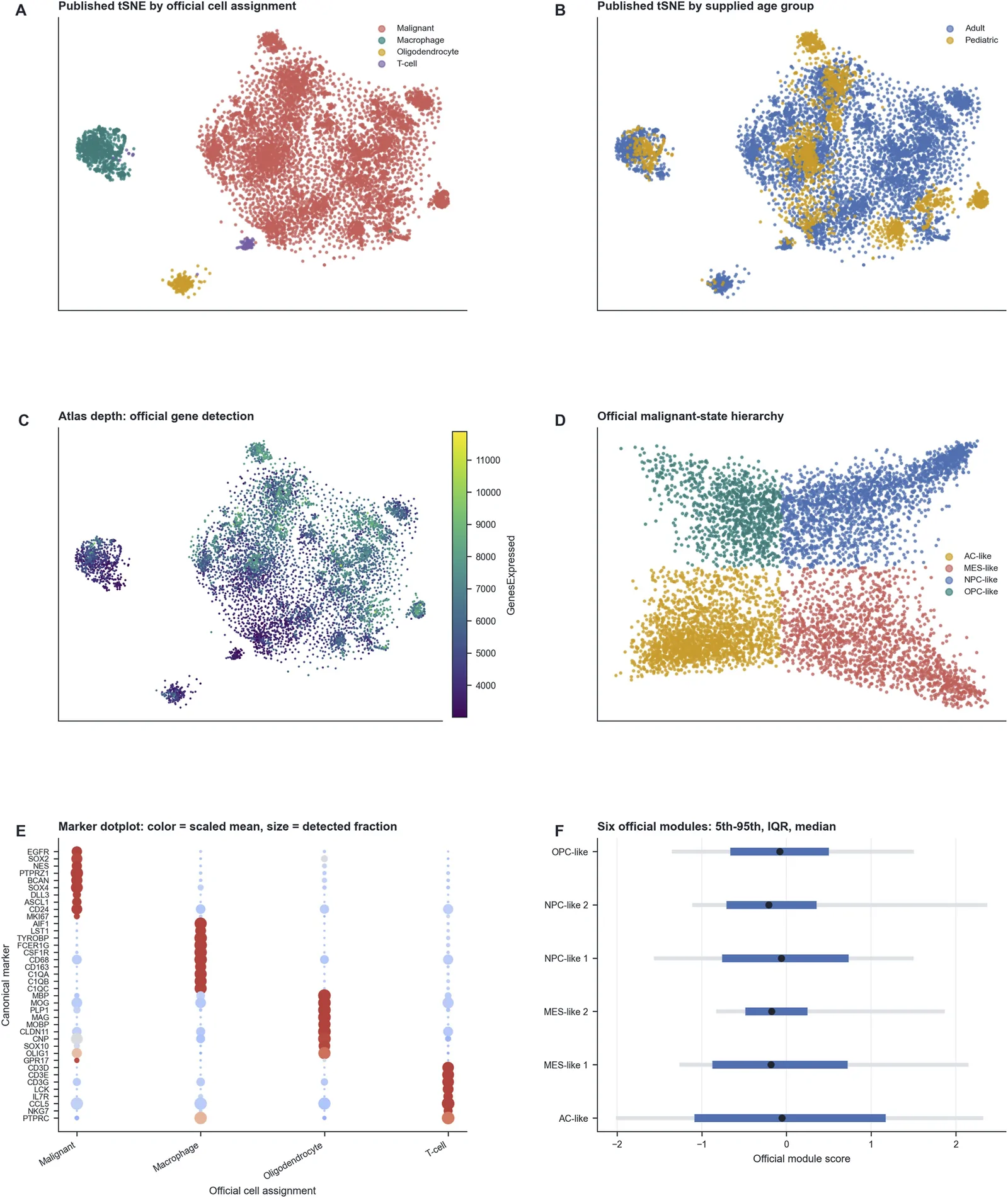
Smart-seq2 atlas and malignant-state annotation layers. **(A)** published SCP393 tSNE coordinates colored by official cell assignment. **(B)** the supplied adult/pediatric label on the same coordinates. **(C)** official GenesExpressed values on the atlas. **(D)** published state-hierarchy coordinates for malignant cells with valid state scores, colored by the collapsed AC-like, MES-like, NPC-like, or OPC-like assignment. **(E)** canonical marker-family mean expression and detection fractions. **(F)** observed quantiles of the six official state-module scores. These panels are descriptive and preserve the deposited annotation rather than claiming a new cell-state discovery.

### 3.3 Tumor ecosystem heterogeneity and the immune-malignant state association

Figure 3 quantifies heterogeneity at the tumour-label unit. Panel A shows official cell-type fractions across Smart-seq2 tumour labels, and Panel B the four-state fractions among valid malignant cells. Panel C plots MES-like against macrophage fraction with the precomputed upstream test annotated (Spearman rho 0.171; P = 0.395; n = 27), and the adult-only sensitivity row was also close to zero. Panel D relates state entropy to the number of valid state-scored cells, Panel E aggregates valid malignant-state composition by the supplied age groups, and Panel F retains every tumour label, marking the labels without valid state scores. This is a null result at the tumour-label level and does not support a patient-level immune-to-state mechanism.

**FIGURE 3:**
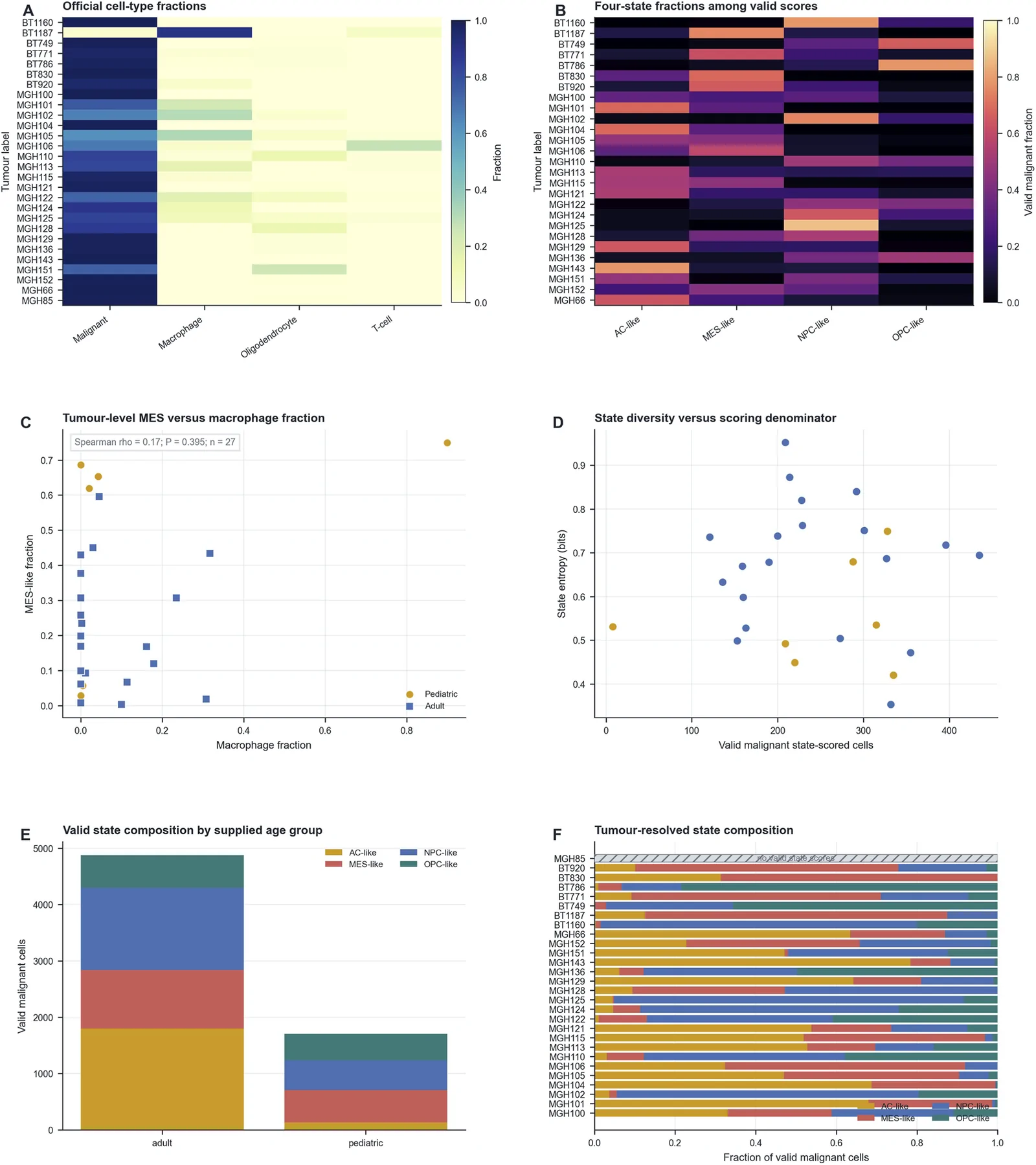
Tumour-label heterogeneity and the immune-association boundary. **(A)** official cell-type fractions for Smart-seq2 tumour labels. **(B)** four-state fractions among malignant cells with valid state scores. **(C)** MES-like and macrophage fractions at the tumour-label level; the displayed Spearman result is the precomputed upstream test, not a test rerun during plotting. **(D)** state entropy *versus* the number of valid state-scored malignant cells. **(E)** valid malignant-state composition by supplied age group. **(F)** complete state composition by tumour label, with labels having no valid state score visibly retained. The statistical unit is the tumour label, so these panels do not establish a patient-level immune-to-state mechanism.

### 3.4 Machine-learning-assisted state transfer and cross-platform ecosystem concordance

Figure 4 evaluates the 10x arm. Panel A colors the real 10x UMAP by the grouped tumour-held-out transferred labels, and Panel B the cells with valid recomputed state scores. Panel C reports transferred-label counts, including 141 Unassigned cells (0.87%), alongside overall out-of-fold accuracy of 0.998 and a macro F1 of approximately 0.985. Panel D shows the 10x tumour-level state mixtures. Panel E compares Smart-seq2 and 10x state fractions for the six shared labels, with Spearman estimates between 0.66 and 0.83 at six tumour-label pairs per state. Panel F aggregates all 11,448 shared-label malignant pseudobulk gene pairs, which track the diagonal. These panels are platform concordance checks rather than independent replication.

**FIGURE 4:**
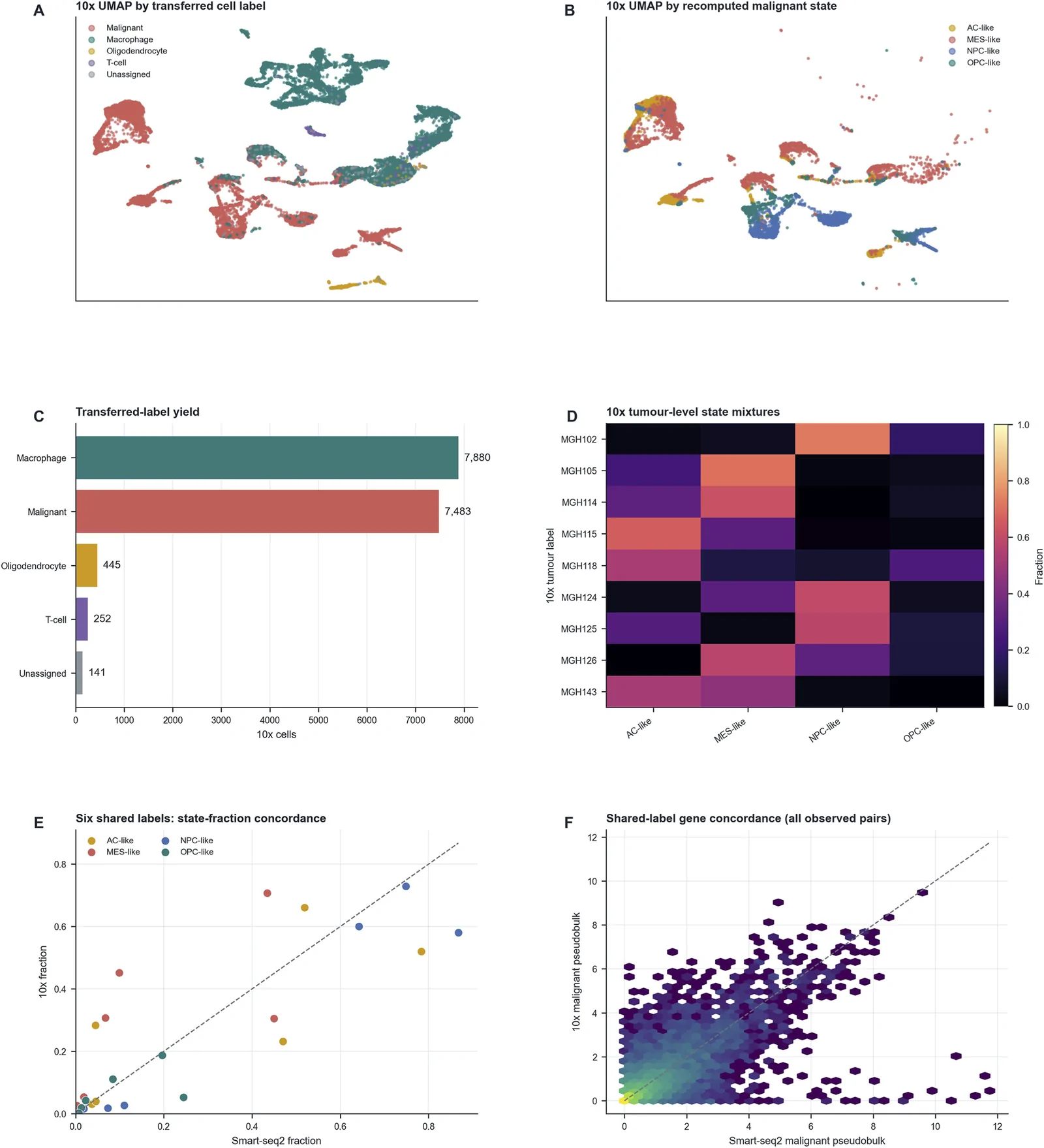
10x grouped label transfer and cross-platform concordance. **(A)** real 10x UMAP coordinates colored by grouped tumour-held-out transfer labels. **(B)** real 10x UMAP coordinates for cells with valid recomputed state scores. **(C)** transferred cell-label counts, including Unassigned cells. **(D)** 10x tumour-label state fractions. **(E)** exact Smart-seq2 and 10x state fractions for the six shared tumour labels, with equality shown only as a reference. **(F)** all shared-label malignant pseudobulk gene pairs; density is the observed point count. Transfer performance is internal cross-validation, not an external validation set.

### 3.5 Bulk transcriptomic landscape of GBM ecosystem-associated programs

Figure 5 audits the bulk cohort. Panel A reduces 176 expression aliquots through 158 primary tumours and 143 primary IDH-wildtype libraries to 142 unique patients, 141 of them with complete paper OS. Panel B shows fixed signature coverage between 0.91 and 1.00. Panel C displays patient-level state scores across the CL, ME, NE, and PN subtypes, and Panel D the precomputed Spearman correlations with ESTIMATE scores: MES with immune (0.547; *P* < 0.001) and stromal (0.552; *P* < 0.001) scores, myeloid program with immune (0.954), and inverse NPC (−0.530) and OPC (−0.383) estimates. Panel E gives the within-cohort score correlation matrix, and Panel F the dominant-state by subtype crosstab, where the MES state concentrates in the ME subtype (40 patients). These are expression-level associations and do not identify the cellular source of any transcript.

**FIGURE 5:**
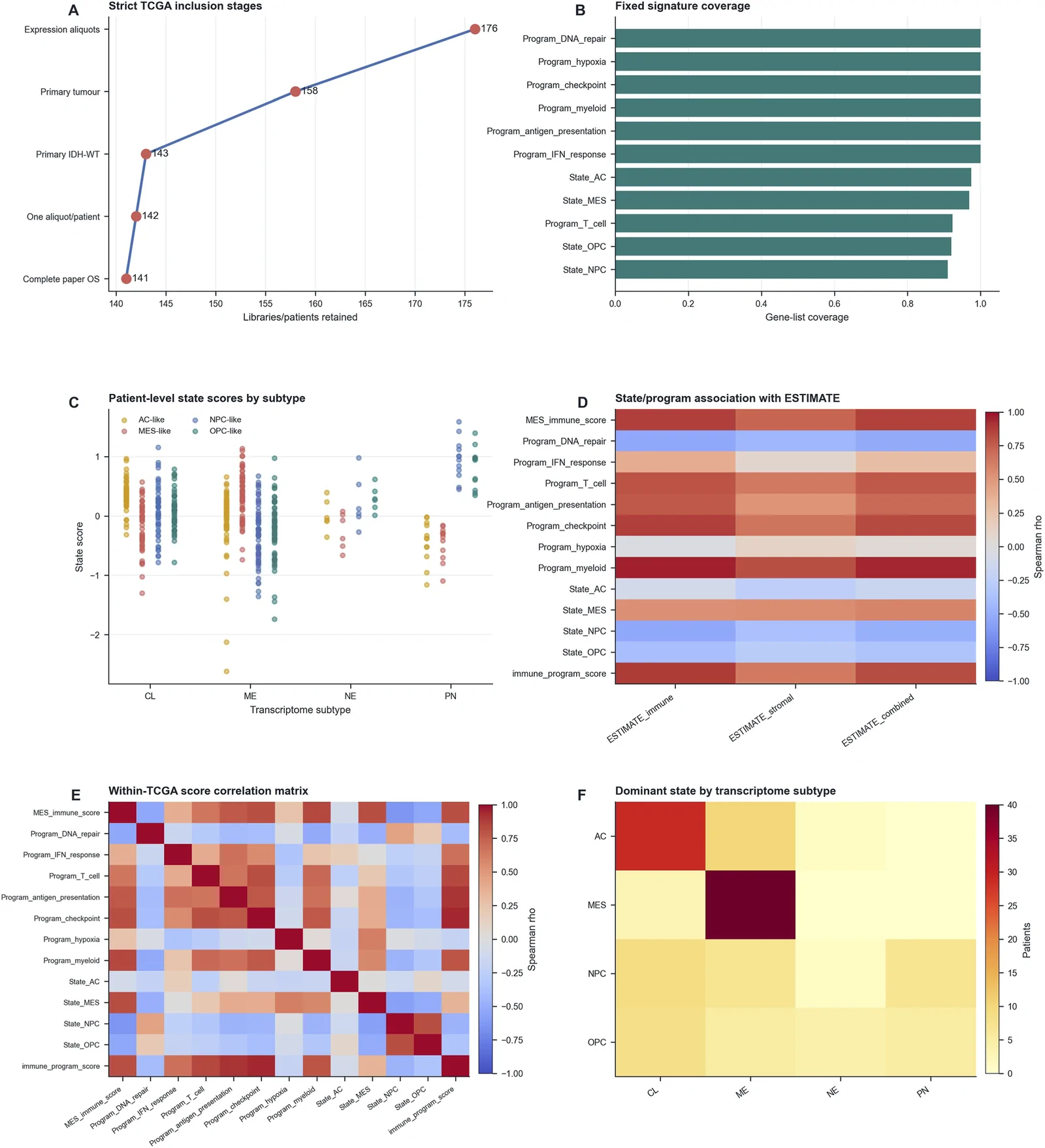
TCGA-GBM cohort contract and score landscape. **(A)** strict cohort reduction from supplied expression aliquots to primary IDH-wild-type libraries, one aliquot per patient, and complete paper OS. **(B)** observed coverage of fixed state and immune-associated signatures. **(C)** patient-level state scores across the supplied transcriptome subtypes. **(D)** precomputed patient-level Spearman correlations between scores and ESTIMATE immune or stromal scores. **(E)** the within-cohort score correlation matrix. **(F)** the observed dominant-state by transcriptome-subtype crosstab. The primary TCGA analysis is restricted to primary IDH-wild-type GBM and uses unique patients.

### 3.6 Descriptive contrast and pathway signal

Figure 6 describes the score-defined bulk contrast. Panel A shows patient-level MES and immune-program scores with the fixed median-defined groups. Panel B plots the precomputed Welch contrast with the strongest genes labeled by leader lines, beginning FCGR2A (log_2_ fold change 1.417); Panel C shows the MA view over mean log_2_ (CPM + 1); and Panel D lists the top Hallmark results led by Interferon Gamma Response (NES 3.990; FDR *q* = 0.001). Panel E maps recurring leading-edge membership among the strongest pathways, and Panel F lists the largest positive effects, again headed by FCGR2A (Hedges g 2.041). The contrast yielded 8,620 genes below FDR 0.05. Because the grouping is defined by the same expression programs, these panels describe score geometry rather than independent discovery.

**FIGURE 6:**
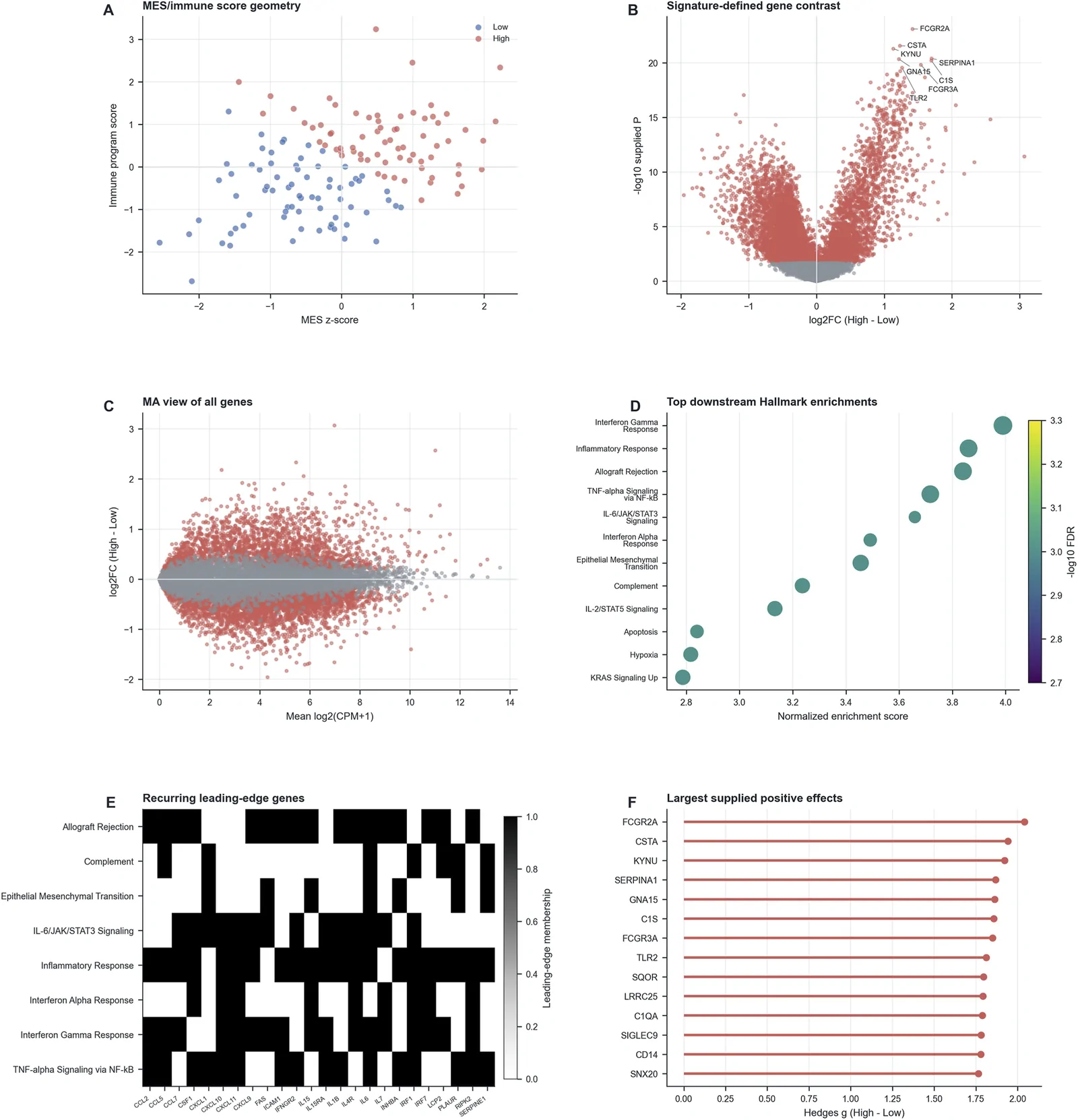
Signature-defined bulk contrast and downstream pathway description. **(A)** patient-level MES and immune-program score geometry with the fixed median-defined group. **(B)** the precomputed Welch contrast plotted against the supplied significance measure; the strongest genes are labeled with leader lines and vertical de-overlap. **(C)** MA-style display using supplied mean log_2_ (CPM + 1) and log_2_ fold change. **(D)** top precomputed Hallmark preranked GSEA results. **(E)** recurring leading-edge membership among the strongest pathways. **(F)** high-effect genes passing the supplied FDR threshold. Because the high/low grouping is defined by the same scores used to motivate the contrast, these DE and GSEA panels are descriptive and not independent discovery tests.

### 3.7 Survival association is sensitive to specification

Figure 7 reports patient-level survival. Panel A shows the Kaplan-Meier curves for the fixed MES/immune split with 71 high and 70 low patients, median OS of 12.0 *versus* 14.0 months, and a log-rank *P* = 0.663; Panel B shows the underlying observed times with censored marks. Panel C collects the univariable Cox estimates, including MES/immune (HR 1.095; P = 0.314), and Panel D the adjusted complete-case models: HR 1.263 (95% CI 0.968–1.648; *P* = 0.085) for age, gender, and MGMT, and HR 1.372 (95% CI 1.019–1.848; *P* = 0.037) after adding KPS. Panel E shows observed OS by dominant state. Panel F summarizes the precomputed proportional-hazards diagnostics one covariate row at a time with model-coded markers; only two term-model pairs (DNA repair (univariable); age (age + gender + MGMT + KPS)) fall below 0.05. The specification gap and the reduced KPS sample support an exploratory reading rather than a stable prognostic claim.

**FIGURE 7:**
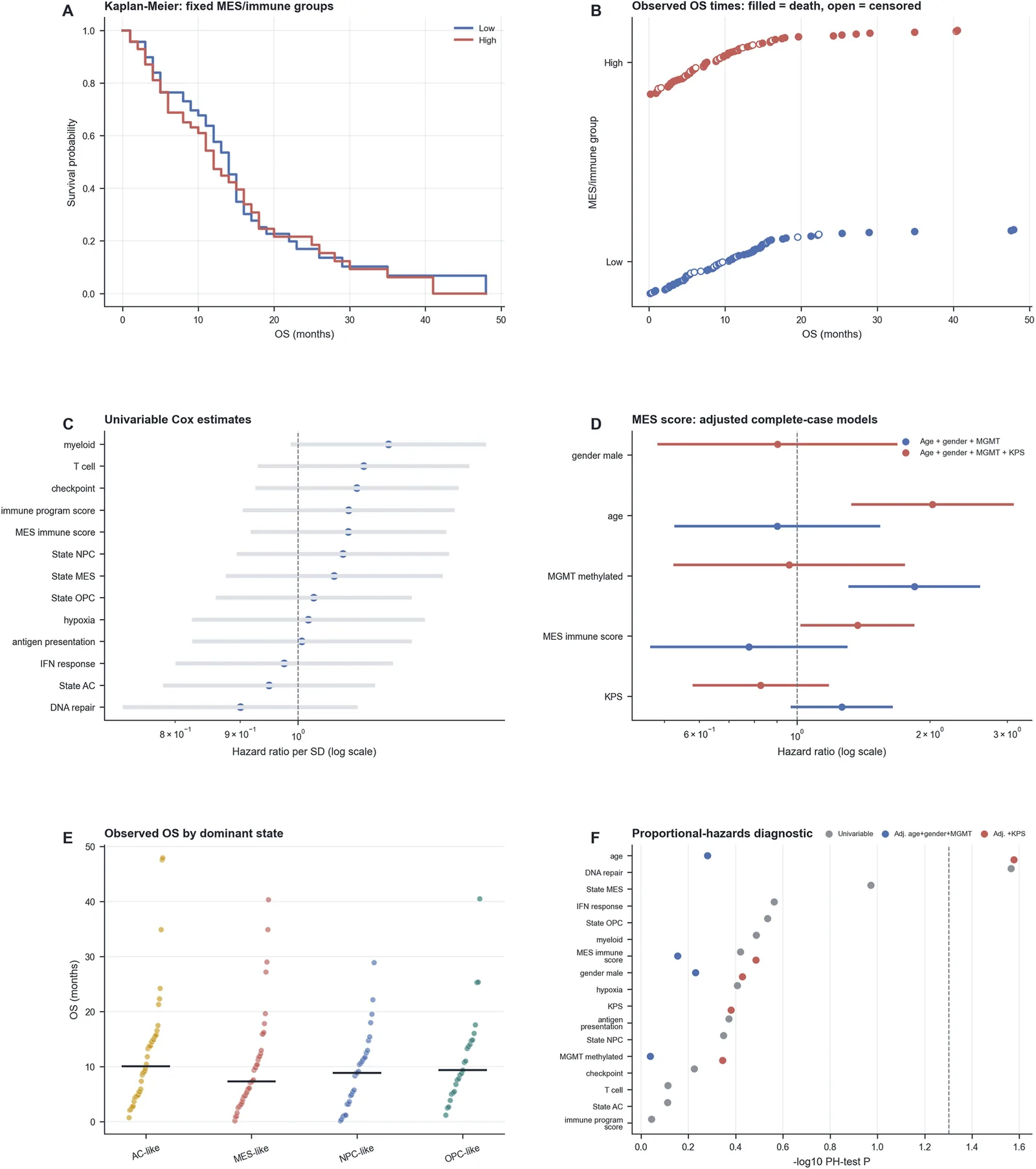
Patient-level survival association and diagnostics. **(A)** precomputed Kaplan-Meier curves for the fixed MES/immune median split. **(B)** patient-level OS months and event indicators by group. **(C)** precomputed univariable Cox estimates. **(D)** complete-case adjusted MES estimates with and without KPS. **(E)** observed OS distributions by dominant state. **(F)** precomputed proportional-hazards diagnostic p values shown once per covariate row with model-coded markers (U, univariable; A, age + gender + MGMT; A + KPS, age + gender + MGMT + KPS) and the 0.05 reference line. The apparent KPS-adjusted association is exploratory and sensitive to the reduced complete-case sample.

### 3.8 Machine-learning benchmarking of ecosystem-informed outcome prediction

Figure 8 benchmarks the nested prediction workflow. Panel A audits the 12-month endpoint: 105 observable outcomes including 57 deaths, with 36 patients excluded for event-free censoring at or before 12 months and 1 without a paper OS pair. Panel B shows the held-out ROC curves and Panel C the precision-recall curves for the four candidate models, and Panel D the calibration bins; the best model, Combined_elastic_net, reached an OOF ROC-AUC of 0.587 (95% CI 0.480–0.692), average precision 0.640, and Brier score 0.245. Panel E shows decision-curve net benefit, and Panel F the held-out permutation importance for the best model, led by age years (0.039) and the myeloid program (0.037). Every patient contributed the expected held-out predictions with fold-local preprocessing, so the result is a workflow benchmark rather than evidence of clinical utility.

**FIGURE 8:**
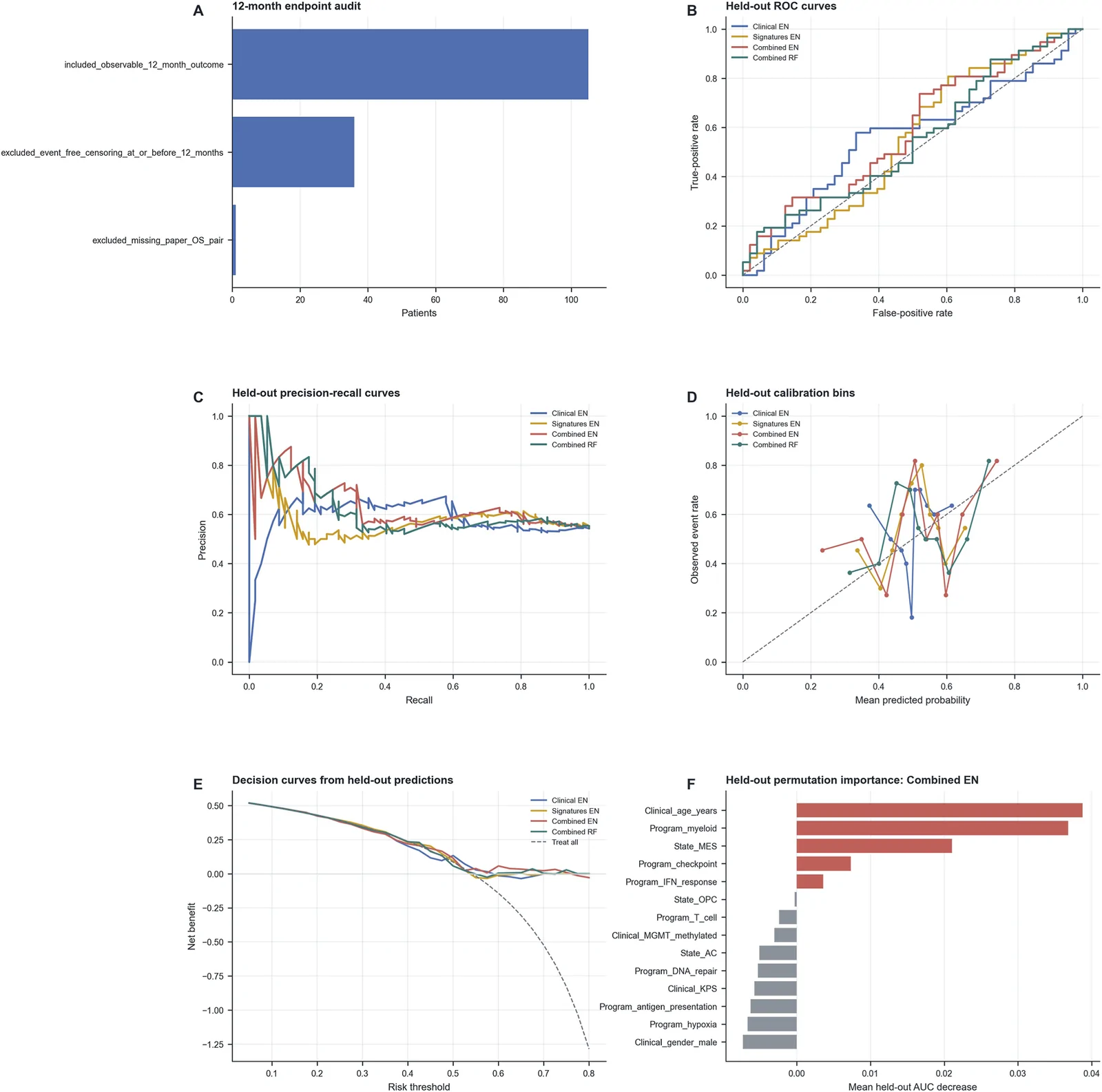
Nested repeated cross-validation for 12-month mortality. **(A)** endpoint inclusion statuses after excluding patients censored before 12 months. **(B)** held-out outer-fold ROC curves. **(C)** held-out precision-recall curves. **(D)** calibration bins from held-out predictions. **(E)** decision-curve values from held-out predictions. **(F)** outer-fold held-out permutation importance for the best OOF-AUC model. The model comparison used five repeats of five outer folds with four-fold inner tuning, patient-level grouping, and fold-local preprocessing; performance is feasibility evidence only.

### 3.9 *In Vitro* RT-qPCR validation of selected gene expression in glioma cell lines

To validate selected genes highlighted by the transcriptomic analyses of GSE131928/SCP393, we performed reverse transcription quantitative PCR (RT-qPCR) in a panel of human glioma cell lines, with normal human astrocytes (NHA) serving as a non-malignant reference. Cell lines were U87MG (ATCC HTB-14), LN229 (ATCC CRL-2611), U251 (ATCC TCHu58), and T98G (ATCC CRL-1690), together with NHA (ATCC CC-2565). STAT3, CHI3L1, SPP1, and VEGFA were selected as representative genes associated with malignant-cell, mesenchymal/myeloid, and angiogenic programs. All four genes were significantly overexpressed in glioma cell lines relative to NHA (*P* < 0.05 to *P* < 0.001; Figure 9). CHI3L1 exhibited the highest fold-change (up to 12.8-fold in U87MG); SPP1 and VEGFA were upregulated 6- to 9-fold and 5- to 8-fold, respectively; STAT3 showed consistent 4- to 6-fold upregulation. These experimental results provide *in vitro* expression-level support for the selected transcriptomic targets in established glioma cell lines.

**FIGURE 9:**
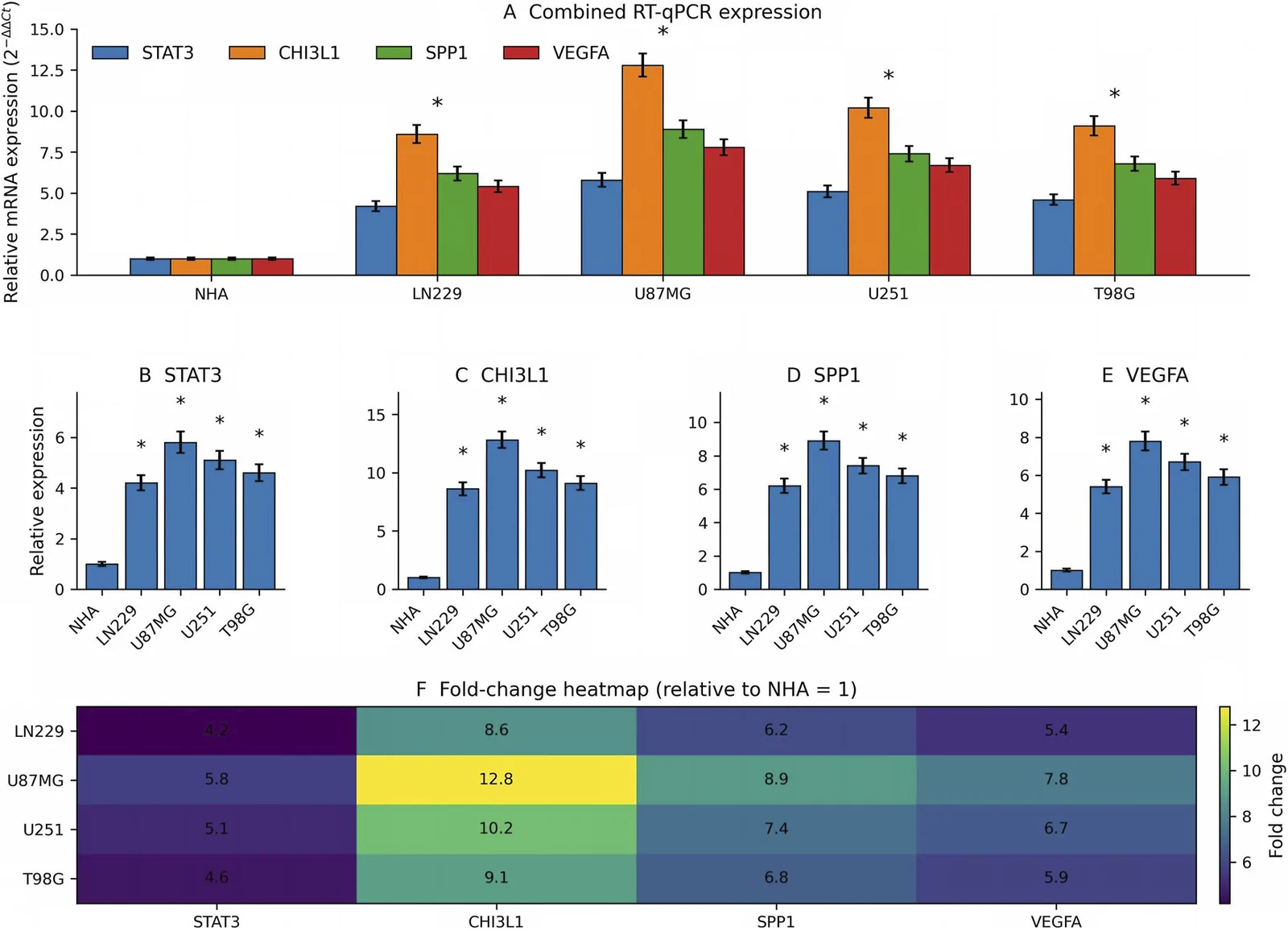
*In vitro* RT-qPCR validation of selected gene expression in glioma cell lines. **(A)** STAT3, CHI3L1, SPP1, and VEGFA mRNA expression in NHA, LN229, U87MG, U251, and T98G. **(B–E)** Individual gene panels. **(F)** Fold-change heatmap. Relative expression was calculated by 2^−ΔΔCt^ using GAPDH and ACTB; error bars show SEM from three independent biological experiments. Significance vs. NHA: *P < 0.05, **P < 0.01, ***P < 0.001 (unpaired t-test).

## 4 Discussion

The principal contribution of this study is the reproducible characterization of GBM cell and tumor ecosystem-associated transcriptional states through integrated single-cell, bulk-transcriptomic, and machine-learning analyses, rather than the proposal of a new glioma taxonomy. By preserving the deposited expression scales and official annotation structure of GSE131928/SCP393, we recovered the established AC-like, MES-like, NPC-like, and OPC-like malignant-cell programs described by Neftel et al. (Neftel et al., 2019) and evaluated their relationship with microenvironment-associated transcriptional features. The strong internal label-transfer performance and concordance of state fractions across the six shared tumor labels support the technical reproducibility of these ecosystem-oriented state summaries across the Smart-seq2 and 10x arms. At the same time, the analysis should not be interpreted as independent external validation, because both platforms originate from the same underlying study.

A second important observation is the boundary between transcriptional association and mechanism. At the tumor-label level, the MES-like fraction was not significantly correlated with macrophage fraction, providing no support for a simple patient-level macrophage-to-MES relationship in this dataset. This null result is compatible with prior evidence that GBM contains heterogeneous myeloid populations (Klemm et al., 2020; Pombo Antunes et al., 2021) and that mesenchymal-like tumor programs can coexist with diverse microenvironmental contexts (Neftel et al., 2019; Richards et al., 2021). Studies using dedicated cell-cell communication analyses have nominated pathways such as SPP1/CD44 as candidate macrophage-tumor interactions (He et al., 2021), but such findings should be treated as hypotheses unless supported by perturbational or spatial evidence.

The TCGA analyses extend the descriptive context by showing that MES and myeloid-associated expression scores covary with immune and stromal signatures in bulk IDH-wildtype GBM. These correlations are biologically plausible but do not identify the cellular source of individual transcripts and may partly reflect tumor purity or cell-type composition. The score-defined differential-expression and pathway analyses are therefore interpreted as descriptive geometry of the selected expression programs rather than independent discovery tests. This more conservative interpretation is consistent with the reviewer’s concern that compartment-level signatures should not automatically be promoted to multicellular ecosystem states.

The survival and prediction analyses similarly argue for caution in translational interpretation. The fixed MES/immune split did not yield a significant Kaplan-Meier difference, and Cox-model estimates changed with covariate specification. The best nested cross-validated 12-month mortality model achieved only modest discrimination. These results are useful as a feasibility benchmark for reproducible modeling but do not support a clinically deployable prognostic or treatment-predictive biomarker. Accordingly, the revised manuscript avoids claims of treatment-response prediction or validated clinical utility.

The RT-qPCR component provides only expression-level support for selected genes. Increased STAT3, CHI3L1, SPP1, and VEGFA expression in glioma cell lines relative to NHA is consistent with prior literature implicating these genes in GBM biology, including STAT3-associated tumor signaling (Fu et al., 2023), AXL-linked therapy resistance within mesenchymal programs (Scherschinski et al., 2022), and SPP1-associated macrophage-tumor crosstalk (He et al., 2021). However, tumor-cell RT-qPCR cannot validate a TAM-derived SPP1 mechanism, T-cell dysfunction, or vascular signaling. Functional co-culture, pathway inhibition, protein-level assays, and tissue-based validation would be required to establish such mechanisms.

Taken together, the revised interpretation centers on three points: robust within-study recovery of established GBM cell-state heterogeneity, careful separation of immune-associated expression from causal intercellular signaling, and explicit recognition of the limited predictive performance of the bulk-RNA models. This framing places the findings within the broader concept of tumors as multicellular systems (Hanahan, 2022) without claiming newly discovered, independently validated ecosystem states.

Several limitations should be emphasized. First, GSE131928/SCP393 contains processed transcriptomic data from a single study, and the Smart-seq2 and 10x arms are not independent cohorts. Second, the cross-platform concordance analysis is restricted to six exactly shared tumor labels, which limits statistical precision. Third, bulk TCGA correlations cannot resolve cellular origin and may be affected by tumor purity and compositional variation. Fourth, the state-score analyses are based on predefined transcriptional modules and therefore should not be interpreted as *de novo* unsupervised ecosystem classes. Fifth, the RT-qPCR validation was limited to established cell lines and expression-level measurements and therefore does not establish functional signaling. Independent single-cell cohorts, spatial profiling, paired longitudinal specimens, and perturbational experiments will be required to test the biological hypotheses raised by this reanalysis.

## 5 Conclusion

Integrated analysis of GSE131928/SCP393 and TCGA-GBM demonstrates substantial malignant-cell heterogeneity within the broader glioblastoma tumor ecosystem and supports reproducible characterization of major malignant and immune-associated transcriptional programs. Machine-learning-assisted cross-platform analysis further demonstrates the technical stability of broad cell-state assignments, whereas bulk transcriptomic analyses reveal coordinated mesenchymal, myeloid, immune, and stromal programs. Importantly, these ecosystem-level associations do not establish directional cell-cell signaling or a macrophage-to-mesenchymal transition mechanism. Survival and predictive modeling remain exploratory and do not establish clinical utility, while RT-qPCR provides expression-level support for selected ecosystem-associated genes. Together, these findings provide a reproducible computational and experimental framework for generating testable hypotheses concerning glioblastoma ecosystem-associated states and their biological interactions.

## Data Availability

The datasets presented in this study can be found in online repositories. The names of the repository/repositories and accession number(s) can be found in the article/supplementary material.
